# Medical student engagement and leadership within a new learning community

**DOI:** 10.1186/1472-6920-10-20

**Published:** 2010-02-26

**Authors:** Mark Bicket, Satish Misra, Scott M Wright, Robert Shochet

**Affiliations:** 1 Division of General Internal Medicine, Johns Hopkins University School of Medicine, Baltimore, MD, USA

## Abstract

**Background:**

Many medical schools are establishing learning communities to foster cohesion among students and to strengthen relationships between students and faculty members. Emerging learning communities require nurturing and attention; this represents an opportunity wherein medical students can become involved as leaders. This study sought to understand issues related to active involvement among students who chose to become highly engaged in a newly developed learning community.

**Methods:**

Between April and June 2008, 36 students who assumed leadership roles within the Colleges Program were queried electronically with open-ended questions about their engagement. Qualitative analysis of the written responses was independently performed by two investigators; coding was compared for agreement. Content analysis identified major themes.

**Results:**

35 students (97%) completed the questionnaire. Motives that emerged as reasons for getting involved included: endorsing the need for the program; excitement with the start-up; wanting to give back; commitment to institutional excellence; and collaboration with talented peers and faculty. Perceived benefits were grouped under the following domains: connecting with others; mentoring; learning new skills; and recognition. The most frequently identified drawbacks were the time commitment and the opportunity costs. Ideas for drawing medical students into new endeavors included: creating defined roles; offering a breadth of opportunities; empowering students with responsibility; and making them feel valued.

**Conclusions:**

Medical students were drawn to and took on leadership roles in a medical school curricular innovation. This example may prove helpful to others hoping to engage students as leaders in learning communities at their schools or those wishing to augment student involvement in other programs.

## Background

In medical school, students encounter robust curricula that often leave little time for personal development and engagement within the academic community. The demanding nature of medical student training has forced educators to reconsider the impact of curriculum on student life [1]. Social isolation, fragmentation of teaching, and limited faculty-learner longitudinal relationships contribute to the burnout and depression that are common among medical students [1-3]. In response to the stresses associated with medical education, some academic medical centers have implemented learning communities - cohorts of students and faculty who collaborate toward common educational and social goals [4-6].

Learning communities contribute positively to stakeholders' perceptions of the educational environment, and in medicine they facilitate increased interaction among medical students as well as between students and faculty members [5]. Medical student involvement in the shaping of the learning community is more likely to result in one that appeals to them. A learning community called 'The Colleges Program' was created in the fall of 2005 at Johns Hopkins University School of Medicine (JHUSOM) to establish longitudinal advising relationships between core faculty and students. The program is composed of 24 core faculty advisors who were chosen for their skill as educators. Faculty and students are organized into one of four colleges. Each faculty advisor is assigned five students from each medical school year. Faculty and students also work closely together during the 'Introduction to Clinical Skills' course in which faculty advisors serve as preceptors for their group of five year one medical students. Goals for the program include enhancing students' career development by consciously fostering camaraderie, networking, and professionalism [6]. During the Colleges Program's development and evolution, a number of students stepped forward and volunteered to take on leadership roles. We conducted this study to characterize the motivators, benefits, and drawbacks for students taking on leadership roles within our learning community as these findings could provide insights to others hoping to activate student engagement in components of the medical school curriculum.

## Methods

### Design and Participants

This study used qualitative analytic methods to purposively examine student leaders enrolled at JHUSOM during the 2007-2008 academic year who volunteered to hold a defined leadership position in the Colleges Program. Leadership roles included planning Colleges-sponsored student programs, coordinating social gatherings, organizing educational events, and directing the peer advising program. These students actively participated in the programs and committed much effort trying to make them successful. To be eligible for inclusion, students had to have spent at least 10 hours working on Colleges related activities in the prior year. All medical students were invited to get involved with this program and those who stepped forward to volunteer were made aware the student leadership roles (described above). Four students had leadership roles but did not dedicate the minimum number of hours and were thus not included in the studied.

### Data Collection

The data collection instrument, comprised primarily of open ended questions (Table 1), was sent electronically using *Survey Monkey*™ between April 30 and June 1, 2008 to the 36 medical student leader informants to understand their perspectives. In addition to the questions related to their engagement and leadership roles, the survey also collected demographic information (age, gender, medical school year). The study was approved by the institutional review board.

**Table 1:** List of the open-ended questions that were asked of the 35 medical student informants leaders

◦ Why did you get involved with Peer Advising and/or the Colleges program?
◦ What benefits do you perceive related to your taking on a committed role within the Colleges program? (How has it helped you or how do you think that it may help you in the future?)
◦ What are the negatives or the drawbacks related to your taking on a committed role within the Colleges program?
◦ Considering other peers who have not gotten involved in a leadership position in Peer Advising and/or the Colleges program, why do you think they have not done so?
◦ If the Colleges or other programs wanted to draw in students to be actively engaged and take on committed roles, how do you think that this could be accomplished most effectively?

### Data Analysis

Demographic data and responses to quantitative questions are presented as means and proportions. The responses from all of the open-ended questions were moved to a master document for analysis. We analyzed written responses to all questions using an "editing analysis style," a qualitative analysis technique in which researchers search for "meaningful units or segments of text that both stand on their own and relate to the purpose of the study" [7]. With this method, the coding template emerges from the data, as opposed to the application of a pre-existing template. Two investigators independently analyzed the transcripts and generated codes to represent informants' statements. Then collaboratively, a coding template was developed. The data was then recoded using this template. In the few instances of discrepant coding, the two investigators successfully reached consensus after reviewing and discussing each other's coding. The authors agreed on representative quotes for each theme.

## Results

### Informant Sampling and Respondent Demographics

Of the 36 medical student leaders sent questionnaires, 35 responded. One female third-year student did not participate. Informant characteristics are summarized in Table 2. Twenty of the responders were male (57%) and the overall mean age was 24 years. Participants were distributed across all class years and a majority (83%) expressed the intent to pursue a career in academic medicine.

**Table 2:** Characteristics of the 35 medical student informants

**Characteristic**		
Women, n (%)	15	(42.8)
		
Age, mean (SD)	24.7	(1.7)
		
Medical School Year, n (%)		
First Year	11	(31.4)
Second Year	11	(31.4)
Third Year	6	(17.2)
Fourth Year	7	(20.0)
		
Plans to pursue a career in academic medicine, n (%)		
Yes	29	(82.9)
No	6	(17.1)
		
College program in which committed role was taken, n (%)		
Peer Advising	7	(20.0)
Social/Community building	14	(40.0)
Both	14	(40.0)
		
Number of hours devoted to Colleges program in the last 12 months, mean (SD)	45.5	(19.6)
		
How invested/committed do you feel toward the goal of making the Colleges program outstanding?, n (%)		
Tremendously	14	(40.0)
A lot	16	(45.7)
Some	4	(11.4)
A little	1	(2.9)
Not at all	0	(0)

In describing their roles within the Colleges, fourteen students (40%) assumed leadership in both the peer advising and social/community building programs. The mean amount of time students devoted to leadership in the Colleges in the prior 12 months was 46 hours. Forty percent of students described being '*tremendously' *committed to making the Colleges program excellent.

### Results of Qualitative Analysis

The comments and stories related by student leaders were categorized into themes that relate to their active involvement in the program. An overview of the themes is presented in Table 3.

**Table 3:** Major themes relating to student engagement

Reasons for getting involved
• Endorsing the need for the program
• Excitement with the start-up
• Wanting to give back
• Commitment to institutional excellence
• Collaborating with talented peers and faculty.

Benefits
• Connecting with others
• Mentoring
• Learning new skills
• Recognition

Drawbacks
• Lack of time
• Opportunity costs

Ideas for drawing in medical students
• Creating defined roles
• Offering a breadth of opportunities
• Empowering students with responsibilities
• Making them feel valued.

### Reasons for getting involved

From the responses of medical student leader informants, five subcategories emerged that explained why the students decided to become actively involved in the learning community. The reasons given can be thought of as the motivations and the expected or hoped advantages.

#### Endorsing the need for the program

Student leaders identified strongly with the goals of the Colleges program and were thrilled that it filled a previously unfulfilled void.

A male first year student stated:

"I think the Colleges program is a great idea. I personally benefited from the system--peer and faculty advising has been great. It is in its incipient stages, and I have enjoyed the opportunity to shape a future legacy at the school."

A 30-year old male fourth year peer advising leader remarked:

"The Colleges program provides an important link between faculty and students, which had been thus far missing, and a way to strengthen or establish inter-class relationships ..."

#### Excitement with the start-up

Many student leaders discussed ideas related to their enthusiasm about being at the beginning of something special. They characterized the early years of the program as a time of potential, novelty, and growth in which they could take part.

A different first year male student who had devoted 30 hours to the program in the last year explained:

"I think that the Colleges program has a great potential of playing a very constructive, uniting, and functional role in students' lives. I felt that by participating, I could contribute towards that goal."

A 29 year old fourth year female student who was 'tremendously' committed to the Colleges wrote:

"I felt a need for better advising programs during my first year and was excited that the administration had plans to address the lack of faculty advising by implementing the Colleges program."

#### Wanting to give back

Medical student leader informants described how a sense of giving back was also at the core of what motivated them to get involved. Specific facets within this domain included the desire to make a difference, to solve problems, and to be beneficent.

A male second-year medical student leader who had devoted 60 hours to the program in the prior year emphasized the significance of giving back:

"I saw peer advising as a way to give back to the med school community, a new program with an identity that we could help create as we participated..."

A different second year medical student peer advising leader shared a sense of reciprocity:

"I wanted to help underclassmen as I had been helped by upperclassmen before."

#### Commitment to institutional excellence

The importance of making the program as successful as possible and of enhancing the school's reputation was referenced by the student leaders.

A male third year medical student who had invested more than 100 hours in the program in the last year reflected on its effect on the institution:

"The program is a great selling point for our school and for our potential students/applicants. When people look for medical schools, they like to compare advising opportunities (at least I did)."

Another dedicated female first year medical student looked to the future impact of the Colleges on both students and the surrounding community:

"I am excited about ... the impact the Colleges program holds in shaping students' Hopkins experience while making a tangible impact on the Baltimore community ... I have been amazed at the difference that a sense of group unity can make in enriching everyone's overall experience."

#### Collaborating with talented peers and faculty

Student leaders described being motivated to work in this environment and having the opportunity to collaborate with capable colleagues, both fellow students and faculty members.

A male second year student who also estimated his time commitment at more than 100 hours in the prior year remarked:

"I got involved primarily because I have a phenomenal advisor. In appreciating what the program had given me, I felt more devoted to it. From there, getting to work with certain classmates and peers reinforced that connection."

A 25 year old female fourth year student with leadership roles in both peer advising and community building commented on how her involvement has impacted relationships with other students and faculty:

"The main reason for me to take on a role in the Colleges program has been to meet students from different classes and to foster inter-class relationships. Another benefit is being able to work with faculty members whom I otherwise would never had met. I feel I am well taken care of and have several mentors..."

#### Benefits

Student leaders listed several actual benefits related to their active involvement in the learning community. Informants noted that their experience with the Colleges program assisted them through: (i) a better defined sense of community with a broader exposure to other students and faculty, (ii) deeper mentoring relationships (both as peer mentor and as mentee), (iii) the ability to learn or refine their own leadership skills, (iv) giving them additional insight about navigating within a large institution, and (v) recognition - both informally from others and in being able to add their contribution to their *curriculum vitae*.

#### Drawbacks

The most common drawback, identified by a majority of respondents, was the amount of time involved in taking on a committed leadership role within the program. Responses from student leaders also indicated that their involvement had 'opportunity costs' in terms of foregoing other activities - including studying and non-educational pursuits.

A female medical student in her final year of medical school summarizes these ideas:

"I think whenever you get involved with any activity; there are always pros and cons... I have sometimes sacrificed personal/academic time to work on colleges/peer advising initiatives - time that could have been spent with my spouse, studying for an exam, doing research. However, I feel that the opportunity to bring about a cultural change at an institution like Hopkins is worth the extra effort on my part and I don't believe I have suffered any long-term negative effects from my involvement...In fact, I think this experience will be useful to me in the future..."

#### Ideas for drawing in medical students

Medical student leader informants also generated several ideas for drawing more medical students into school or faculty-initiated endeavors. Their ideas were organized around the following ideas: (i) creating defined positions for students with specific responsibilities within a logical organizational structure, (ii) providing a diverse array of opportunities for students, (iii) empowering student leaders with specific and meaningful responsibilities, and (iv) implementing steps to make students feel appreciated, respected, and valued.

About these matters, two different female students in the graduating class explained:

"The most effective way to accomplish this to students would be to have them become invested in the process somehow. If students can take on ownership, and feel like they have a little piece of the pie, if you will, and that what they do as students will make a tangible difference then I feel like they will become engaged."

"*If people feel that what they bring to the table is important and special, and will not otherwise be provided if they are absent, they are more likely to become actively engaged, keep to deadlines despite other work-pressures, and contribute productively to their colleges and the program in general."*

## Discussion

In this study, medical students describe motivations, benefits, and drawbacks associated with their voluntary assumption of leadership roles within a new learning community. The emergent themes that explain the reasons that they opted to roll up their sleeves and get involved may have been predicted based on motivational theory [8]. Motivation has been defined as "the power to move or excite individuals to action" [9]. Four of the motivation themes may best be understood in reflecting upon the students' desires to help - others (*wanting to give back, endorsing the need for the program*), the school (*commitment to institutional excellence*), and themselves (*collaboration with talented peers and faculty*). The last explanation for their assumption of a committed leadership role can be attributed to the entrepreneurial spirit of these students - *excitement with the start-up*.

Reflecting upon learning communities in the context of organizational culture may provide insight as to why individuals seek out responsibility. Results from a study performed at successful organizations showed that dedicated employees gave answers that were comparable to the motivators and benefits listed by our committed medical students: opportunities for personal development (*learning new skills*), working with "winners" (*collaborating with talented peers/faculty*), the opportunity to solve problems (*endorsing the need for the program*), recognition of work (*recognition*), and appropriate compensation (*recognition*) [10]. The culture of an organization is made up of the assumptions, values, and norms of the members and their behaviors [11]. Thus, imparting change within a medical school's learning community must include not only altering structures and processes, but also drawing in the student body so as to transform the culture.

There is a fair amount of literature documenting the value of learning communities in higher education [12,13]. Boyer, the former President of the Carnegie Foundation for the Advancement of Teaching, may have most eloquently described the manner in which the learning community can shape the vision for the educational institution by creating a sense of wholeness in education through 6 pillars of shared values: openness, caring, purpose, discipline, justice, and celebration [12]. Uniting a medical school around such principles would be transformative. Although there are few studies describing the merits of learning communities within medical schools, some of the postulated and observed benefits have included networking between students and faculty members, leadership development, and guidance with career planning [5]. To achieve such benefits, medical student engagement as leaders within the learning community is likely to be critical.

Students often come to medical school with robust resumes of leadership in their former collegiate and home communities [14]. However, the academic experience of medical school is intense and may influence students to use their discretionary time for recovery outside of the medical school community. Enticing medical students into optional programs is also difficult because of the stage of life of these driven young adults who have many competing obligations and interests. Medical learning communities or other programs that need genuine student engagement may be well served to consider the themes that emerged related to *'drawing in' *medical students. Creating defined roles, offering a breath of opportunities, empowering students with real responsibility, and genuinely valuing the students and showing it may be instructive to others seeking student buy-in and leadership. Foregoing involvement in the learning community can represent a missed opportunity to leave a meaningful legacy at one's medical school, and disengagement may contribute to a student's social isolation [15]. Further, learning communities within medical schools can explicitly promote student leadership as a core value of the medical profession [16].

Several limitations of this study should be considered. First, this study relied exclusively on self-report. However, this is considered to be the most direct approach for understanding attitudes and beliefs. Second, this study is limited to a small number of medical students in one learning community at a single academic institution and thus our findings may not apply to other institutions. We sampled all possible informants so the issue of thematic saturation and ending data collection early was not necessary to consider. Students who might volunteer for leadership opportunities at other medical schools may be different and may have different perspectives about the benefits and drawbacks. Finally, one student leader declined participation and it is possible that her perspectives may have been different.

## Conclusion

The results of this study shed light onto medical student engagement and leadership within a learning community. The implementation and growth of learning communities in medical schools presents unique opportunities for many groups, particularly students. When medical students become engaged in leadership roles within the learning community, both the students and the learning community are to gain.

## Competing interests

The authors declare that they have no competing interests.

## Authors' contributions

All authors conceived the idea, chose the study design, and developed survey. MB and SW performed the qualitative analysis, but SM and RS were involved by reviewing and providing feedback on the coding template. All authors participated in writing paper and approved the final manuscript.

## Pre-publication history

The pre-publication history for this paper can be accessed here:

http://www.biomedcentral.com/1472-6920/10/20/prepub
